# Evaluation of the Level of Psychological Distress in Construction Workers during the COVID-19 Pandemic in Southern Spain

**DOI:** 10.3390/healthcare12121224

**Published:** 2024-06-19

**Authors:** Carlos Gómez-Salgado, Juan Carlos Camacho-Vega, Regina Allande-Cussó, Carlos Ruiz-Frutos, Mónica Ortega-Moreno, Jorge Martín-Pereira, Israel Macías-Toronjo, Blanca Prieto-Callejero, Juan Jesús García-Iglesias, Javier Fagundo-Rivera, Juan Gómez-Salgado

**Affiliations:** 1 School of Doctorate, University of Huelva, 21007 Huelva, Spain; 2 Department of Building Construction II, Higher Technical School of Building Engineering, University of Seville, 41012 Sevilla, Spain; 3 Department of Nursing, Faculty of Nursing, Physiotherapy and Podiatry, University of Seville, 41009 Sevilla, Spain; rallande@us.es; 4 Department of Sociology, Social Work and Public Health, Faculty of Labour Sciences, University of Huelva, 21007 Huelva, Spain; 5 Safety and Health Postgraduate Programme, Universidad Espíritu Santo, Guayaquil 092301, Ecuador; 6 Department of Economy, Faculty of Labour Sciences, University of Huelva, 21007 Huelva, Spain; 7 Department of Rehabilitation, Fremap Huelva, 21001 Huelva, Spain; 8 Department of Nursing, University of Huelva, 21007 Huelva, Spain; 9 Centro Universitario de Enfermería Cruz Roja, University of Seville, 41009 Sevilla, Spain

**Keywords:** COVID-19, construction industry, psychological distress, occupational safety and health, mental health, construction workers, workplace mental health

## Abstract

The COVID-19 pandemic posed a major challenge for construction companies, which were confronted with the need to prevent the enormous negative socio-psychological impact of the pandemic on their employees. The aim of this study was to evaluate the level of psychological distress among construction workers in an advanced phase of the COVID-19 pandemic in Andalusia, southern Spain. For this, a cross-sectional descriptive study was conducted using online questionnaires with data on sociodemographic variables and employment situation, COVID-19 pandemic-related data, and Goldberg’s General Health Questionnaire (GHQ-12). A total of 860 questionnaires from all provinces of Andalusia, Spain, were collected between March and May 2022. Descriptive statistical analyses and non-parametric Mann–Whitney U and Chi-squared tests were performed, followed by logistic regression analysis. The incidence of psychological distress was higher among women, individuals under 43 years of age, those with a family income below EUR 1200, participants whose working conditions had been affected by the pandemic, those who had not received adequate means or specific training to protect themselves from infection, those who had experienced symptoms, those who had suffered side effects after vaccination, and those who had been hospitalised. The logistic regression analysis predicted the occurrence of psychological distress in this study by the effect of the pandemic on mental/emotional well-being, the working conditions affected during the pandemic, health-related variables, and the age of the worker. The correctly classified percentage was 75.1%. Assessing psychological distress in construction sectors may allow for the identification of vulnerable groups or even help to reduce the number of errors in daily practice and potential risks of occupational injury or illness.

## 1. Introduction

The construction sector accounts for 13% of the world’s gross domestic product (GDP) and employs a low-skilled workforce compared to other productive sectors. Another feature that differentiates it from other jobs is that many activities are impossible to perform virtually [1,2]. In countries such as the UK, it accounts for 10% of employment [3], and it is a sector with high occupational accident rates worldwide [4]. In Spain, in 2023, workers in the construction sector represented 6.3% of the employed population and, specifically, 6.1% in Andalusia (204,100 workers) [5].

While research into mental health in the workplace in general and in the construction sector in particular is still in its early stages, it is known that suicide rates among low-skilled workers in the construction sector were considerably higher than the national rate in the UK [6], and other studies carried out before the COVID-19 pandemic revealed that two out of five construction workers suffer from depression and anxiety and, more seriously, three out of five workers have problems with alcohol consumption [7]. The most commonly used instruments to assess the mental health of workers in the construction industry are scales for depression, anxiety, and stress [8].

The mental health of construction workers has recently come to the forefront of occupational health and safety research, but the effectiveness of workplace interventions at an organisational level has been found to be limited [9]. Even before the COVID-19 pandemic, differences had been found between builders and supervisors in terms of psychosocial risks at work and their impact on mental health, with the former having a higher prevalence of the need to recover after work and more frequent distress, depression, or post-traumatic stress disorder [10].

For all the above said, this workforce faces a high-risk and mentally stressful work environment [11] and has one of the highest levels of workplace stressors, all of which has been exacerbated by the pandemic [12]. The role of work stress in favouring both unsafe behaviours and the level of safety participation has been shown to predispose to accidents yet not in terms of levels of safety compliance [13]. Workplace safety and health in construction is one of the seven challenges identified in the construction sector [14], which was affected by three types of safety-related stressors, role ambiguity, role conflict, and interpersonal safety conflict, where self-efficacy mediated between these three stressors and safety participation [15]. Workplace behaviours have been associated with job satisfaction and innovative behaviour at work, which is of particular interest at a time of increasing unemployment rates in this population group [16].

The severe acute respiratory syndrome coronavirus 2 (SARS-CoV-2), as the cause of COVID-19, has led to a major health crisis that brought about economic recession and psychological insecurity worldwide [16]. Conveniently, the assessment of previous epidemics has made it possible to identify effects such as anxiety, depression, or increased psychiatric morbidity [17,18,19].

In 2022, Spain faced the seventh wave of the COVID-19 pandemic, marked by a peak in incidence on January 21st with 3418 cases per 100,000 inhabitants over fourteen days. This increase began in November 2021 and accelerated in December. However, starting on January 21st, the incidence started to decrease rapidly, falling by 33% in fifteen days. The severity of cases during this wave was significantly lower than in previous ones, with hospitalisation, ICU admission, and mortality rates between 10 and 22 times lower. The high vaccination coverage in Spain, with 90.8% of the population over 11 years of age vaccinated and 91% of those over 60 years of age with booster doses, was key in reducing the vulnerability and severity of cases [20].

Construction workers suffered the highest mortality rates during the COVID-19 pandemic [21]. This was despite the fact that in the US the collective adapted to perform some tasks by videoconferencing, like in other countries, in order to reduce the transmission of the disease [22], and specific guidelines were developed to manage and prevent the disease among workers [23].

Assuming that the impact on the sector may vary across countries, negative effects have been found, such as high unemployment rates in the US [24] and supply chain or material price increases, but also positive ones, such as the awareness of the need to incorporate digitalisation [25]. Therefore, a report from the Spanish Association of Major Construction Companies of Spain describes that the economic crisis caused by COVID-19 in Spain led to the collapse of up to 63% of public work contracts tendered and awarded; in this regard, the sector had to face a loss of nearly EUR 2.5 billion in tenders and close to EUR 3 billion in contracts [26].

The pandemic posed a major challenge for companies, as it required adapting working conditions, both technically and physically, to prevent the massive negative socio-psychological impact [27].

In this context, the present study represents the first assessment of the impact of the COVID-19 pandemic on the psychological distress of construction workers in Spain. Thus, the aim of this study was to assess the level of psychological distress (PD) among construction workers in an advanced phase of the COVID-19 pandemic in Andalusia, southern Spain. It also sought to identify personal and occupational variables determining this level of PD, which could be used to implement preventive measures in future health crises and other types of crises.

## 2. Materials and Methods

### 2.1. Design

A cross-sectional observational study employing electronic survey instruments was carried out.

### 2.2. Population and Sample

The research population comprised individuals employed in the Andalusian sector in 2023, totalling 204,100 workers, with women constituting 8.03% of the population [5]. A sample size of 383 participants was initially established, with a confidence level of 95%, a precision of 3.5%, and an adjustment for potential loss of 10%. However, the final sample size was 860 subjects.

A non-probabilistic convenience sample was used. Questionnaires were received from all provinces of Andalusia, although a higher proportion was received from Seville and Huelva. The sample of workers analysed contained a higher proportion of men (81.7%), in line with the current sex distribution in the construction sector.

Several companies were approached to participate in this study, and none refused to do so. Companies were asked to send the survey link to all their employees, regardless of their level of awareness of the prevalence of PD among their employees. Workers accessed the questionnaire mainly through their own mobile devices, although in some cases company-provided computers were used. The online questionnaire link was sent via email to companies based in Andalusia, trade union organisations, and workers’ associations in the sector who agreed to participate in this study and disseminate the questionnaire among their workers, between March and May of 2022. Information about the project, including a QR code and a printed poster to facilitate dissemination among workers, was attached to the emails.

To access the questionnaire items, participants were required to first access the information sheet and provide informed consent. Without this consent, it was not possible to proceed with answering the items.

### 2.3. Instruments

The previously validated Emotional Impact Questionnaire COVID-19 (EIQ COVID-19) tool [28], which incorporates questions adapted from previous research [29], was used and expanded with industry-specific data tailored to the construction sector. This instrument encompassed sociodemographic characteristics such as age, sex, geographic location, employment status (self-employed, full-time, part-time, redundancies, unemployed), occupational classification (managerial, skilled, intermediate management, labourer, administrative, or custodial staff), nature of work, construction site type (residential, industrial, or civil), work environment (outdoor or indoor), income adequacy, household size, dwelling size in square meters, and usage of workplace dining facilities (yes, no, only in low attendance, non-existent, or closed during the pandemic).

Furthermore, personal information pertinent to the COVID-19 pandemic was collected, encompassing aspects such as diagnosis, isolation experience, severity of illness, hospitalisation history, vaccination status and associated side effects, availability and utilisation of preventive measures, received training, perception of workplace safety, and the impact of the pandemic on work-related activities.

The variable ‘Pandemic effect on mental/emotional well-being’ had ‘No’ as a reference, and the alternative options were ‘Yes’ or ‘Possibly’.

As a strong predictor of morbidity [30] which has been included in previous epidemics [31] and COVID-19 studies [29], self-perceived health status was also measured. The variable ‘Overall health and physical condition’ took values between 1 and 10.

To assess psychological distress, the Goldberg General Health Questionnaire, GHQ-12, was used, comprising 12 items and offering 4 response options, with a total scoring range of 0 to 12 points [32]. The threshold applied was ≥3, aligning with the threshold used in Spanish national surveys [33]. The GHQ-12 has demonstrated strong reliability across various studies, with Cronbach’s alphas ranging from 0.82 to 0.86 [32]. The internal consistency index achieved was α = 0.905.

### 2.4. Data Analysis

A univariate descriptive analysis was conducted, obtaining frequencies, means, and standard deviations based on the type of variable, as well as a bivariate analysis. Prior to the bivariate analysis, the result of the Kolmogorov–Smirnov test was obtained, which indicated the non-normality of the sample with a value of *p* < 0.05. For the bivariate analysis, contrast statistics such as the Mann–Whitney U test and the Chi-squared test were used to determine whether there were any statistically significant differences between the median scores and the classified score (GHQ < 3 or GHQ ≥ 3) for psychological distress and the other study variables, respectively.

A binary logistic regression analysis was also performed to build a predictive model for the presence of psychological distress and the other variables. The model was built by introducing significant variables identified using the Wald method, with the objective of building a simple and robust model. To assess the model’s adequacy, various goodness-of-fit measures were used, including the Hosmer–Lemeshow test, percentage of correctly classified values, sensitivity, and specificity. Statistical significance tests were used to determine the inclusion of variables, so odds ratios were estimated and confidence intervals were provided for this measure of association. All analyses were carried out using SPSS 26.0 statistical software (IBM, Armonk, NY, USA).

### 2.5. Ethics Statement

The 2013 Declaration of Helsinki (Fortaleza meeting, Brazil) was observed in this study. Written informed consent was obtained from the participants for the confidential use and treatment of the data in accordance with the Spanish Data Protection and Digital Rights Act of 2018. Participants were informed that their data would be duly safeguarded by the research team. A favourable opinion was obtained from the Regional Research Ethics Committee (PI_036-20).

## 3. Results

### 3.1. Sociodemographic Characteristics of the Sample

The group of construction workers surveyed was predominantly male (81.7%), with a mean age of 42.7 years and 61.2% of them living with a partner. In total, 75.5% of them reported a family income of more than EUR 1200 per month, and 43.5% stated that their income was sufficient to make ends meet. In terms of employment status, 78.6% of individuals had a full-time contract, 12.5% were self-employed, and 5.9% were part-time employees. To a lesser extent, there were workers who were unemployed at the time (2.8%) or who were still in the exceptional situation created during the pandemic to reduce redundancies through temporary redundancy procedures, which were partly subsidised by public funds (0.6%) (Table 1).

In terms of the degree of responsibility at work, the highest percentage was found among labourers (47.7%), and 40.0% were managers and qualified staff or held middle management positions, while 12.3% were administrative or cleaning staff.

According to the questionnaires, building works (57.1%) were the most frequently reported type of construction work, followed by civil works (18.5%) and industrial works (14.3%). Additionally, 41.9% of workers were engaged in outdoor work, and 24.2% used company canteens (Table 1).

### 3.2. Psychological Distress and Personal or Employment-Related Variables

The prevalence of PD (GHQ < 3) among workers was 29.2%, with a higher incidence in women (37.7%) compared to men (27.3%) (*p* = 0.010). Participants with PD were younger (mean = 41.2; SD = 10.4) than those without PD (mean = 43.4; SD = 10.3) (*p* = 0.006). Subjects with PD had lower levels of health and a worse physical condition (*p* < 0.001).

In terms of marital status, individuals who cohabited with a partner had a lower risk of developing PD (25.9%) than those who did not (34.4%) (*p* = 0.007). The presence of financial resources to make ends meet was associated with the development of PD, with those who reported having such resources being less likely to develop PD (24.3%) compared with those who did not (32.9%) (*p* = 0.006). It can be seen that respondents with a family income of less than EUR 1200 had a greater likelihood of experiencing PD (35.1%) compared to respondents with an income of more than EUR 1200 (27.3%), *p* = 0.030 (Table 2).

For the variables size of dwelling, type of construction site, degree of responsibility, working outdoors–indoors, or the use of canteens, no differences were found regarding the development of PD.

### 3.3. Psychological Distress and Data Related to the COVID-19 Pandemic

A higher percentage of PD (39.7%) was found among workers whose working conditions had been affected during the pandemic than among those whose working conditions had not been affected (17.4%), *p* < 0.001. In companies where protective measures such as masks, gloves, gels, and goggles had not been provided by the managing staff, 36.7% had PD, compared to 26.8% (*p* = 0.005) in companies where such measures had been provided. Additionally, workers who had not received specific training on COVID-19 were more likely to develop PD, with a prevalence of 33.1%, compared to 25.8% in those who had received such training (*p* = 0.020).

The perception of safety and protection from infection while performing job duties was found to be associated with lower rates of PD. Specifically, 21.7% of those who felt completely safe had PD, compared to 35.1% of those who felt somewhat safe and 63.2% of those who felt not at all safe (*p* < 0.001). Having had side effects after COVID-19 vaccination was found to be associated with developing PD, with 34.4% of those who had had side effects and 25.2% of those who had not reporting PD (*p* = 0.003). The presence of COVID-19 symptoms was associated with a higher percentage of PD (33.0%) compared to those without symptoms (26.6%) (*p* = 0.042).

It was found that 44.2% of workers who reported that the situation experienced during the COVID-19 pandemic had negatively affected their mental/emotional well-being had developed PD at a much higher rate than those who reported the opposite (9.7%) (*p* < 0.001) (Table 3).

### 3.4. Variables Determining the Development of Psychological Distress among Workers in the Construction Sector

The variables that seem to be most predictive of the level of PD among construction workers are the following: ‘the effect of the pandemic on mental/emotional well-being’, OR = 6.182 (CI95%: 4.204, 9.089); ‘working conditions affected during the pandemic’, OR = 2.281 (CI95%: 1.628, 3.196); ‘variables related to health and physical condition’, OR = 0.797 (CI95%: 0.744, 0.854); and ‘age of the worker’, OR = 0.980 (CI95%: 0.969, 0.992). These variables predict 75.1% of the effect, an R^2^ = 0.428, a sensitivity of 44.2%, and a specificity of 87.9% (Table 4).

## 4. Discussion

Women in this study, a minority in the sector and in this sample, presented higher percentages of PD than men, something observed in most published studies both in the general population [34,35] and in the healthcare sector [36]. Consistent with previous studies, younger respondents, in this study those under 43 years of age, were the most likely to develop PD [35,37].

Individuals living alone, whose figures increased during the pandemic, were found to be more likely to develop PD [35]. This is consistent with the results in the present study in which a higher percentage of PD was found among workers living without a partner, with no statistically significant difference being observed based on the number of cohabitants. In other studies, a great heterogeneity and susceptibility towards developing PD was observed in different periods of the pandemic, where only those classified with a ‘chronic’ profile of living alone experienced a significant change, yet this variation was not found in the other groups [38].

PD in this study population (29.2%) was still very high but much lower than the levels found in non-healthcare workers during the first phase of the pandemic (67.3%) [39]. This discrepancy suggests that although there was a 33% reduction in the incidence of cases during the data collection period [20], psychological distress remained a significant condition among construction workers. Possible explanations for this sustained high level of PD despite the decrease in incidence could include lingering effects of the pandemic on mental health, such as increased stress and anxiety due to ongoing uncertainty, economic pressure, and changing working conditions [40]. Additionally, it is important to consider the unique challenges already faced by construction workers, such as the physical demands of the job, potential exposure to hazardous materials, and irregular employment patterns, which could contribute to persistent psychological distress even as overall COVID-19 cases declined.

Another aspect to consider is the cut-off point established for determining PD, i.e., GHQ-12 ≥ 3. It may be necessary to raise this cut-off point to ≥5 in order to better discriminate the affected population and not be faced with too high a percentage of the population that would hinder the identification of those people who require intervention priorities. It has been found in a UK study that the percentage of disorders found was much higher when using the GHQ-12 as a screening tool (with a cut-off of 4), with a prevalence of 52.8%, than when using a diagnostic interview as a tool (13.7%) [41]. Further stressors that may lead to high levels of PD may persist after the COVID-19 pandemic has been overcome.

Workers’ income is a conditioning variable for developing PD, and thus, the negative association between family well-being and PD was stronger among those with lower incomes. This in particular justifies exploring how inequality in family resources may affect mental health to a greater extent [35,42]. In this study, a family income of less than EUR 1200 or not having enough money to make ends meet was found to be associated with the development of PD.

The present study did not detect an association between living in a small dwelling and a higher incidence of PD, which in previous studies had been attributed to the difficulty of taking preventive measures against infection [43,44]. It is possible that this discrepancy is due to the fact that these studies were carried out in early phases of the pandemic, whereas the data in the present study were obtained at a later stage.

A correlation was found between the incidence of PD and the use of preventive measures in the workplace [45]. Also, lack of compliance with the use of such preventive measures was associated with male sex, young age, low income, low perceived effectiveness of preventive measures, or high perceived cost of compliance with such measures, among others [46]. The present study found that PD may be associated with the level of preventive measures provided by companies, the perception that working conditions had been affected during the pandemic, that they did not feel safe and protected from infection in their workplace, or that they had not received specific training to prevent infection. In this regard, it has been highlighted that companies in the construction sector can play a role in reducing the level of PD among their staff during health crises by ensuring that workers have sufficient preventive measures and by providing specific training in their use. In this sense, as the levels of physical and emotional stress undermine compliance with safety standards among construction workers [12], effective interventions are needed to benefit workers, organisations, and the economy in general [47].

No statistically significant association was found between different employment situations and PD nor between full-time and part-time contract type. This is probably due to the complexity of contract types in the construction sector, which during the pandemic was supplemented by new types of contracts to reduce dismissals, such as temporary redundancy procedures. Other studies not specific to the construction industry have found that self-employed workers had developed a higher level of PD compared to those employed in public or private companies [39].

Having had symptoms during the pandemic or negative effects after vaccination was associated with a higher prevalence of PD, but no differences were found between those who had been vaccinated and those who had not. Perceived health is often included in censuses and used in epidemiological studies [30] as an indicator to consider in research. It is therefore not surprising that the proportion of workers who responded that the COVID-19 pandemic had negatively affected their mental/emotional well-being was taken as an important variable in predicting the development of PD.

It has been reported in previous studies that construction workers may have poorer mental health than workers in other sectors and that the COVID-19 pandemic may have worsened this situation, as observed in Australia [48]. Beyond that, the most noticeable effects on mental health have been reported, mainly, among under-skilled construction workers and those with the lowest salaries [49]. Also, working longer hours per week compared to the norm in other productive sectors has been suggested to influence the mental health of workers in the construction sector [50]. The results of this study can be used to identify individual aspects, such as age or profession, preventive measures adopted by companies, or organisational factors that may be the source of empirical research on intervention strategies, as suggested by previous studies [51].

A limitation of this study is that it has a cross-sectional observational design that only reports perceptions at the time of this study. Thus, it does not allow cause-and-effect relationships to be established but, on the contrary, provides very valuable data on the final stages of the pandemic and identifies associations that may lead to hypotheses to be tested in subsequent research with different designs.

Sample collection was not randomly carried out, and the sex ratio was asymmetrical, not corresponding to the distribution of the Spanish population but to the distribution of the studied sector. These factors were balanced by a large and representative sample from all provinces, accounting for the sex variable when analysing the sample.

In addition, when using self-administered questionnaires, researchers must rely on the veracity of the data provided by the respondents. Furthermore, the use of online surveys may introduce biases due to limited access to technology and the internet, potentially excluding certain demographic groups. It should be noted that some of the variables for which a significant difference in terms of the association with PD was observed are based on the results of the bivariate analysis, although several of these variables did not remain significant in the multivariate model. The final model has a high specificity, which is useful for reducing false positives. On the other hand, its sensitivity is relatively low, which means that it does not detect all cases of psychological distress and results in many people in need of help not being identified by the model. Another limitation is that the working conditions differed greatly from one respondent to another, and this might be a confounding factor in this study.

Finally, several measures are suggested to reduce the number of construction workers suffering from post-traumatic stress disorder (PTSD). These measures include the implementation of preventive measures and specific training to mitigate the impact of the pandemic on the mental/emotional well-being of workers. Additionally, the importance of improving working conditions affected during the pandemic is highlighted, as workers who experienced changes in these conditions were found to have higher rates of PTSD. The active response of companies in the sector to health crises such as the one caused by COVID-19 has also been identified as a relevant strategy. This response involved the implementation of appropriate preventive measures and the provision of psychological support to workers. Furthermore, the significance of socio-economic and personal factors that can serve as protective factors against psychological distress is emphasised, thus enhancing workers’ resilience. Finally, it is recommended that PTSD assessment in sectors such as the construction industry during a pandemic be employed to identify vulnerable groups and implement targeted preventive measures with the objective of reducing the incidence of occupational injury or illness and improving overall workplace safety.

## 5. Conclusions

In this study, the logistic regression analysis predicted the occurrence of PD by the effect of the pandemic on mental/emotional well-being, the working conditions affected during the pandemic, health-related variables, and the age of the worker. The correctly classified percentage was 75.1%. In fact, those workers whose working conditions had been affected during the pandemic had higher rates of PD than those who did not state such an effect.

The response of construction companies to a major health crisis such as the one caused by COVID-19 was found to be associated with the level of psychological distress experienced by their employees. This was determined through the provision of preventive measures and specific training to workers by these companies. Additionally, socio-economic and personal variables that may prevent psychological distress were also identified.

PD assessment in sectors such as construction during a pandemic can help to identify vulnerable groups. In addition, it can help to reduce the number of errors in daily practice by identifying workplaces where there is a potentially higher risk of occupational injury or disease.

## Figures and Tables

**Table 1:** Sociodemographic characteristics.

	Cases (%)
Sex	
Male	703 (81.7%)
Female	154 (17.9%)
Intersex	3 (0.3%)
Age [mean (SD)]	42.7 (10.4)
Marital status	
Married or cohabiting	526 (61.2%)
Other situations	334 (38.8%)
Approximately, how many square metres (m^2^) does your dwelling have?	
0–50 m^2^	28 (3.3%)
51–75 m^2^	154 (17.9%)
76–100 m^2^	312 (36.3%)
101–125 m^2^	170 (19.8%)
126–150 m^2^	105 (12.2%)
More than 150 m^2^	91 (10.6%)
Do you consider that your income is sufficient to make ends meet?	
Yes	374 (43.5%)
No or depending on the month	486 (56.5%)
What is the total monthly family income?	
Between EUR 0 and 1200	211 (24.5%)
More than EUR 1200	649 (75.5%)
Employment situation	
Self-employed	104 (12.1%)
Full-time employee	676 (78.6%)
Part-time employee	51 (5.9%)
Temporary redundancy procedure	5 (0.6%)
Unemployed	24 (2.8%)
Degree of responsibility	
Managers and skilled workers	209 (24.3%)
Intermediate management	135 (15.7%)
Manual workers	410 (47.7%)
Others (administration staff, cleaning…)	106 (12.3%)
Type of construction work	
Building work	491 (57.1%)
Civil work	159 (18.5%)
Industrial work	123 (14.3%)
More than one type of work	86 (10.0%)
Does not know/say	1 (0.1%)
Construction site	
Outdoors	360 (41.9%)
Indoors (of buildings, facilities…)	500 (58.1%)
Use of staff canteen	
Yes	208 (24.2%)
No	612 (71.2%)
Other cases	40 (4.6%)

**Table 2:** Psychological distress and associated personal or employment data.

	NO GHQ < 3		YES GHQ ≥ 3			
	Cases	%	Cases	%	Statistical (Effect Size)	*p*-Value
Total	609	70.8%	251	29.2%		
Age [Median (IQR)]	43.4 (15)		41.2 (16)		66,022.5 (0.099) **	0.006
Overall health and physical condition [Median (IQR)] *	8 (2)		8 (1)		61,608.50 (0.156) **	<0.001
Sex						
Male	511	72.7%	192	27.3%	6.551 (0.087) ***	0.010
Female	96	62.3%	58	37.7%		
Intersexual	2	66.7%	1	33.3%	-	-
Marital status						
Married or cohabiting	390	74.1%	136	25.9%	7.269 (0.092) ***	0.007
Other situations	219	65.6%	115	34.4%		
Approximately, how many square metres (m^2^) does your dwelling have?						
0–75 m^2^	122	67.0%	60	33.0%	1.597 (0.043) ***	0.206
More than 75 m^2^	487	71.8%	191	28.2%		
Employment status						
Full-time employee/self-employed	558	71.5%	222	28.5%	4.010 (0.069) ***	0.045
Part-time employee/temporary redundancy procedure	33	58.9%	23	41.1%		
Unemployed	18	75.0%	6	25.0%	-	-
Do you consider your income sufficient to make ends meet?						
Yes	283	75.7%	91	24.3%	7.546 (0.094) ***	0.006
No or depending on the month	326	67.1%	160	32.9%		
What is the total monthly family income?						
Between EUR 0 and 1200	137	64.9%	74	35.1%	4.685 (0.074) ***	0.030
More than EUR 1200	472	72.7%	177	27.3%		
Degree of responsibility						
Managers and qualified staff	139	66.5%	70	33.5%	4.802 (0.075) ***	0.187
Mid-level management	99	73.3%	36	26.7%		
Labourer	301	73.4%	109	26.6%		
Other (administrative staff, cleaning staff, …)	70	66.0%	36	34.0%		
Type of construction work						
Building work	348	70.9%	143	29.1%	6.950 (0.090) ***	0.073
Civil work	124	78.0%	35	22.0%		
Industrial work	82	66.7%	41	33.3%		
More than one type of work	55	64.0%	31	36%		
DK/DR	0	0%	1	100%		
Construction site						
Outdoors (open air)	254	70.6%	106	29.4%	0.020 (0.005) ***	0.888
Indoors (buildings, facilities, etc.)	355	71.0%	145	29.0%		
Use of staff canteen						
Yes	153	73.6%	55	26.4%	1.075 (0.036) ***	0.300
No	427	69.8%	185	30.2%		
Other cases	29	72.5%	11	27.5%	-	-

DK/DR: Don’t know/respond; IQR: Interquartile Range; * Scoring from 1 to 10; ** Mann–Whitney U test; *** Chi-squared test.

**Table 3:** Psychological distress and COVID-19-related data.

	NO GHQ < 3		YES GHQ ≥ 3		Chi-Squared Test	
	Cases	%	Cases	%	Statistical	*p*-Value
Total	609	70.8%	251	29.2%		
Have you been diagnosed with COVID-19?						
Yes	247	68.4%	114	31.6%	1.724 (0.045)	0.189
No	362	72.5%	137	27.5%		
Has anyone in your circle been diagnosed with COVID-19?						
Yes	535	71.2%	216	28.8%	0.516 (0.025)	0.472
No	74	67.9%	35	32.1%		
Has anyone in your circle died from COVID-19?						
Yes	74	69.2%	33	30.8%	0.162 (0.014)	0.687
No	535	71.0%	218	29.0%		
Have you been isolated because you have had the disease or contact with a person tested positive?						
Yes	334	68.7%	152	31.3%	2.361 (0.052)	0.124
No	275	73.5%	99	26.5%		
Have you had mild symptoms?						
Yes	231	67.0%	114	33.0%	4.148 (0.069)	0.042
No	378	73.4%	137	26.6%		
Have your working conditions been affected by the pandemic?						
Yes	273	60.3%	180	39.7%	51.539 (0.245)	<0.001
No	336	82.6%	71	17.4%		
Have your managers or your company provided and do they provide you with the necessary protective measures to avoid contagion (masks, gloves, gels, goggles)?						
Yes	457	73.2%	167	26.8%	7.932 (0.097)	0.005
No	143	63.3%	83	36.7%		
Other	9	90%	1	10%	-	-
Did you receive or have you ever received specific training on COVID-19 disease (transmission routes, self-protection measures, warning signs) organised by your managers or your company?						
Yes	343	74.2%	119	25.8%	5.417 (0.080)	0.020
No	255	66.9%	126	33.1%		
Other (self-employed, mid-level, …)	11	64.7%	6	35.3%	-	-
In general, do you feel safe and protected from infection in the performance of your work duties?						
Yes, totally safe	360	78.3%	100	21.7%	39.653 (0.215)	<0.001
Somewhat safe	235	64.9%	127	35.1%		
No, not at all safe	14	36.8%	24	63.2%		
Have you been vaccinated against COVID-19?						
Yes	600	71.0%	245	29.0%	0.864 (0.032)	0.353
No	9	60.0%	6	40.0%		
Have you had any side effects following vaccination?						
Yes	244	65.6%	128	34.4%	8.652 (0.10)	0.003
No	365	74.8%	123	25.2%		
Do you think that the situation experienced during the COVID-19 pandemic has negatively affected your mental/emotional well-being?						
Yes	272	55.85%	215	44.15%	121.616 (0.376)	<0.001
No	337	90.35%	36	9.65%		

**Table 4:** Variables determining the development of psychological distress among workers in the construction sector.

	Odds Ratio (Confidence Interval at the 95% Level)
Effect of the pandemic on mental/emotional well-being (Ref. NO)	6.182 ** (4.204, 9.089)
Working conditions affected by the pandemic (Ref. NO)	2.281 ** (1.628, 3.196)
Health and physical condition	0.797 * (0.744, 0.854)
Age	0.980 ** (0.969, 0.992)
Sensitivity (%)/specificity (%)	44.2/87.9
Correctly classified percentage	75.1%
R^2^	0.428
Hosmer–Lemeshow test	χ^2^ = 5.138 (*p* = 0.743)
Omnibus test	χ^2^ = 329.678 (*p* < 0.001)

* *p* < 0.005; ** *p* < 0.001.

## Data Availability

The original contributions presented in this study are included in this article; further inquiries can be directed to the corresponding authors.

## References

[1] Araya F. (2021). Modeling the spread of COVID-19 on construction workers: An agent-based approach. Saf. Sci..

[2] Olanrewaju A., AbdulAziz A., Preece C.N., Shobowale K. (2021). Evaluation of measures to prevent the spread of COVID-19 on the construction sites. Clean. Eng. Technol..

[3] Mitchell A. (2020). CLC Statement on Payment and Contracts.

[4] Meng X., Chan A.H.S. (2022). Cross-Regional Research in Demographic Impact on Safety Consciousness and Safety Citizenship Behavior of Construction Workers: A Comparative Study between Mainland China and Hong Kong. Int. J. Environ. Res. Public Health.

[5] Instituto de Estadística y Cartografía de Andalucía (2023). Encuesta de Población Activa—Visualización del Número Ocupados por Sector de Actividad. https://www.juntadeandalucia.es/institutodeestadisticaycartografia/epa/visualizacion-actividad.htm.

[6] Burki T. (2018). Mental health in the construction industry. Lancet Psychiatry.

[7] Lim S., Chi S., Lee J.D., Lee H.J., Choi H. (2017). Analyzing psychological conditions of field-workers in the construction industry. Int. J. Occup. Environ. Health.

[8] Chan A.P.C., Nwaogu J.M., Naslund J.A. (2020). Mental Ill-Health Risk Factors in the Construction Industry: Systematic Review. J. Constr. Eng. Manag..

[9] Greiner B.A., Leduc C., O’Brien C., Cresswell-Smith J., Rugulies R., Wahlbeck K., Abdulla K., Amann B.L., Pashoja A.C., Coppens E. (2022). The effectiveness of organisational-level workplace mental health interventions on mental health and wellbeing in construction workers: A systematic review and recommended research agenda. PLoS ONE.

[10] Boschman J.S., van der Molen H.F., Sluiter J.K., Frings-Dresen M.H. (2013). Psychosocial work environment and mental health among construction workers. Appl. Ergon..

[11] He C., Jia G., McCabe B., Chen Y., Sun J. (2019). Impact of psychological capital on construction worker safety behavior: Communication competence as a mediator. J. Saf. Res..

[12] Liang H., Yang W., Liu T., Xia F. (2022). Demographic Influences on Perceived Stressors of Construction Workers during the COVID-19 Pandemic. Int. J. Environ. Res. Public Health.

[13] Wang D., Wang X.Q., Xia N.N. (2018). How safety-related stress affects workers’ safety behavior: The moderating role of psychological capital. Saf. Sci..

[14] Sierra F. (2021). COVID-19, Main challenges during construction stage. Eng. Constr. Archit. Manag..

[15] Ye G., Xiang Q., Yang L., Yang J., Xia N., Liu Y., He T. (2022). Safety Stressors and Construction Workers’ Safety Performance: The Mediating Role of Ego Depletion and Self-Efficacy. Front. Psychol..

[16] Ren T., Cao L., Chin T. (2020). Crafting Jobs for Occupational Satisfaction and Innovation among Manufacturing Workers Facing the COVID-19 Crisis. Int. J. Environ. Res. Public Health.

[17] Bish A., Michie S. (2010). Demographic and attitudinal determinants of protective behaviours during a pandemic: A review. Br. J. Health Psychol..

[18] Rubin G.J., Amlôt R., Page L., Wessely S. (2009). Public perceptions, anxiety, and behaviour change in relation to the swine flu outbreak: Cross sectional telephone survey. BMJ.

[19] Van Bortel T., Basnayake A., Wurie F., Jambai M., Koroma A.S., Muana A.T., Hann K., Eaton J., Martin S., Nellums L.B. (2016). Psychosocial effects of an Ebola outbreak at individual, community and international levels. Bull. World Health Organ..

[20] Government of Spain Real Decreto 115/2022, de 8 de Febrero, por el que se Modifica la Obligatoriedad del uso de Mascarillas durante la Situación de Crisis Sanitaria Ocasionada por el COVID-19. Boletín Oficial del Estado, n 34, 09 de Febrero de 2022. https://www.boe.es/eli/es/rd/2022/02/08/115.

[21] Office for National Statistics U.K. (2020). Coronavirus (COVID-19) Related Deaths by Occupation, England and Wales: Deaths Registered up to and Including 20 April 2020. https://www.ons.gov.uk/peoplepopulationandcommunity/healthandsocialcare/causesofdeath/bulletins/coronaviruscovid19relateddeathsbyoccupationenglandandwales/deathsregistereduptoandincluding20april2020.

[22] Nnaji C., Jin Z., Karakhan A. (2022). Safety and Health Management Response to COVID-19 in the Construction Industry: A Perspective of Fieldworkers. Process Saf. Environ. Prot..

[23] Scottish Government (2020). Coronavirus (COVID-19): Construction Sector Guidance. https://www.scottishfuturestrust.org.uk/publications/documents/construction-sector-covid-19-guidance.

[24] Bureau of Labor Statistics (BLS), USA (2023). Unemployed Persons by Industry, Class of Worker, and Sex. https://www.bls.gov/web/empsit/cpseea31.htm.

[25] Ayat M., Kang C.W. (2023). Effects of the COVID-19 pandemic on the construction sector: A systemized review. Eng. Constr. Archit. Manag..

[26] Spanish Association of Major Construction Companies of Spain El impacto de la COVID-19 en la construcción. https://www.musaat.es/blog/el-impacto-de-la-covid-19-en-la-construccion/.

[27] Conversano C., Marchi L., Miniati M. (2020). Psychological Distress among Healthcare Professionals Involved in the COVID-19 Emergency: Vulnerability and Resilience Factors. Clin. Neuropsychiatry.

[28] Gómez-Salgado J., Andrés-Villas M., Domínguez-Salas S., Díaz-Milanés D., Ruiz-Frutos C. (2020). Related Health Factors of Psychological Distress during the COVID-19 Pandemic in Spain. Int. J. Environ. Res. Public Health.

[29] Wang C., Pan R., Wan X., Tan Y., Xu L., Ho C.S., Ho R.C. (2020). Immediate psychological responses and associated factors during the initial stage of the 2019 coronavirus disease (COVID-19) epidemic among the general population in China. Int. J. Environ. Res. Public Health.

[30] Eriksson I., Unden A.L., Elofsson S. (2001). Self-rated health. Comparisons between three different measures. Results from a population study. Int. J. Epidemiol..

[31] Main A., Zhou Q., Ma Y., Luecken L.J., Liu X. (2011). Relations of SARS-related stressors and coping to Chinese college students’ psychological adjustment during the 2003 Beijing SARS epidemic. J. Couns. Psychol..

[32] Goldberg D.P., Gater R., Sartorius N., Ustun T.B., Piccinelli M., Gureje O., Rutter C. (1997). The validity of two versions of the GHQ in the WHO study of mental illness in general health care. Psychol. Med..

[33] Rocha K.B., Pérez K., Rodríguez-Sanz M., Borrell C., Obiols J.E. (2011). Propiedades psicométricas y valores normativos del general health questionnaire (GHQ-12) en población general española. Int. J. Clin. Health Psychol..

[34] Matud M.P., Zueco J., Díaz A., Del Pino M.J., Fortes D. (2022). Gender differences in mental distress and affect balance during the first wave of COVID-19 pandemic in Spain. Curr. Psychol..

[35] McCallum S.M., Calear A.L., Cherbuin N., Farrer L.M., Gulliver A., Shou Y., Dawel A., Batterham P.J. (2021). Associations of loneliness, belongingness and health behaviors with psychological distress and wellbeing during COVID-19. J. Affect. Disord. Rep..

[36] Arias-Ulloa C.A., Gómez-Salgado J., Escobar-Segovia K., García-Iglesias J.J., Fagundo-Rivera J., Ruiz-Frutos C. (2023). Psychological distress in healthcare workers during COVID-19 pandemic: A systematic review. J. Saf. Res..

[37] Best R., Strough J., Bruine de Bruin W. (2023). Age differences in psychological distress during the COVID-19 pandemic: March 2020–June 2021. Front. Psychol..

[38] Bayes-Marin I., Cabello-Toscano M., Cattaneo G., Solana-Sánchez J., Fernández D., Portellano-Ortiz C., Tormos J.M., Pascual-Leone A., Bartrés-Faz D. (2023). COVID-19 after two years: Trajectories of different components of mental health in the Spanish population. Epidemiol. Psychiatr. Sci..

[39] Ruiz-Frutos C., Ortega-Moreno M., Allande-Cussó R., Domínguez-Salas S., Dias A., Gómez-Salgado J. (2021). Health-related factors of psychological distress during the COVID-19 pandemic among non-health workers in Spain. Saf. Sci..

[40] Tham W.W., Sojli E., Bryant R., McAleer M. (2021). Common Mental Disorders and Economic Uncertainty: Evidence from the COVID-19 Pandemic in the U.S. PLoS ONE.

[41] Scott H.R., Stevelink S.A.M., Gafoor R., Lamb D., Carr E., Bakolis I., Bhundia R., Docherty M.J., Dorrington S., Gnanapragasam S. (2023). Prevalence of post-traumatic stress disorder and common mental disorders in health-care workers in England during the COVID-19 pandemic: A two-phase cross-sectional study. Lancet Psychiatry.

[42] Chen B., Gong W., Lai A.Y.K., Sit S.M.M., Ho S.Y., Yu N.X., Wang M.P., Lam T.H. (2023). Family context as a double-edged sword for psychological distress amid the COVID-19 pandemic with the mediating effect of individual fear and the moderating effect of household income. Front. Public Health.

[43] Allande-Cussó R., García-Iglesias J.J., Miranda-Plata R., Pichardo-Hexamer R., Ruiz-Frutos C., Gómez-Salgado J. (2022). Social Determinants of Health in the COVID-19 Pandemic Context of the Migrant Population Living in Settlements in Spain. Int. J. Public Health.

[44] Nyashanu M., Simbanegavi P., Gibson L. (2020). Exploring the Impact of COVID-19 Pandemic Lockdown on Informal Settlements in Tshwane Gauteng Province, South Africa. Glob. Public Health.

[45] Ruiz-Frutos C., Delgado-García D., Ortega-Moreno M., Duclos-Bastías D., Escobar-Gómez D., García-Iglesias J.J., Gómez-Salgado J. (2021). Factors Related to Psychological Distress during the First Stage of the COVID-19 Pandemic on the Chilean Population. J. Clin. Med..

[46] Pollak Y., Shoham R., Dayan H., Gabrieli-Seri O., Berger I. (2022). Background and concurrent factors predicting non-adherence to public health preventive measures during the chronic phase of the COVID-19 pandemic. J. Public Health.

[47] Nwaogu J.M., Chan A.P., Hon C.K., Darko A. (2019). Review of global mental health research in the construction industry: A science mapping approach. Eng. Constr. Archit. Manag..

[48] Dickson R. (2023). What’s it going to take? Lessons learned from COVID-19 and worker mental health in the Australian construction industry. Constr. Manag. Econ..

[49] King T.L., Lamontagne A.D. (2021). COVID-19 and suicide risk in the construction sector: Preparing for a perfect storm. Scand. J. Public Health.

[50] Crook D., Tessler A. (2021). The Cost of Doing Nothing Report [Online]. BIS Oxford Economics and the Culture in Construction Taskforce. https://cict.mymedia.delivery/wp-content/uploads/2021/05/The-Cost-of-Doing-Nothing-Report.pdf.

[51] Newaz M.T., Giggins H., Ranasinghe U. (2022). A Critical Analysis of Risk Factors and Strategies to Improve Mental Health Issues of Construction Workers. Sustainability.

